# Organic Fertilization and Biostimulant Application to Improve Yield and Quality of Eggplant While Reducing the Environmental Impact

**DOI:** 10.3390/plants14060962

**Published:** 2025-03-19

**Authors:** Luigi Giuseppe Duri, Roberta Paradiso, Ida Di Mola, Eugenio Cozzolino, Lucia Ottaiano, Roberta Marra, Mauro Mori

**Affiliations:** 1 Department of Agricultural Sciences, University of Naples Federico, 80055 Naples, Italy; luigigiuseppe.duri@unina.it (L.G.D.); ida.dimola@unina.it (I.D.M.); lucia.ottaiano@unina.it (L.O.); robmarra@unina.it (R.M.); mauro.mori@unina.it (M.M.); 2 Council for Agricultural Research and Economics (CREA), Research Center for Cereal and Industrial Crops, 81100 Caserta, Italy; eugenio.cozzolino@crea.gov.it

**Keywords:** *Solanum melongena* L., compost, digestate, plant-based biostimulant, microbial biostimulant

## Abstract

Environmental sustainability is a crucial issue in modern agriculture and special attention needs to be paid to soil health preservation. Eggplant (*Solanum melongena* L.) cultivation implies the supply of relevant quantities of chemical fertilizers, since the crop has high nutrient requirements. This study investigated the combined effects of two common organic amendments—compost and digestate—and two types of biostimulant—a plant-based product and a microbe-based product—on fruit production and quality of eggplant, to highlight the potential synergistic effects of fertilization and biostimulation. The experiment was carried out in a Mediterranean greenhouse in the winter/spring period, assessing early and total marketable yield and fruit qualitative traits (firmness, color, nitrogen, ascorbic acid, carotenoid and phenol content, and antioxidant activity). Results showed that the fertilization strategy significantly influenced plant productivity, with digestate promoting the early fruitification and mineral fertilizers resulting in a higher total yield. Biostimulants, particularly the microbial type, improved the fruit quality in terms of carotenoid content and antioxidant activity. These findings highlight the potential benefits of combining organic amendments with biostimulants in eggplant cultivation, enhancing the economic value of the product through the increase in the early production and fruit nutraceutical value while realizing sustainable practices.

## 1. Introduction

Among the sustainable development goals of the Agenda 2030, endorsed by governments of the 193 member countries of the United Nations and approved by the General Assembly, the planet’s health and the sustainability of production and consumption processes require wide attention.

Accordingly, environmental sustainability has gained importance in many aspects of daily life during the last years. In agriculture, this concept is mainly associated with the rational use of pesticides and chemicals directly applied on plants. However, it is often neglected that sustainability should start from the soil health. Indeed, in the Mediterranean basin, intensive farming has resulted in several negative effects, including the reduction in soil organic matter (hence, in soil fertility) with consequent soil degradation [1].

The enhancement of soil health can be achieved through proper management strategies. For instance, as stated by Amsili et al. [2], a positive impact can be obtained through those practices, which positively influence carbon and nutrient cycling [3].

According to Clapp et al. [4], a notable increase in the use of organic amendments occurred in response to the decline in organic matter in agricultural soils, with compost and digestate among the most common products. Composting is an effective and ecologically friendly method for recovering and valuing organic biomasses, including bio-wastes, within the agro-food chain [5]. Digestate is a derivative of biodegradable residues and by-products of livestock and agro-industrial sectors, and it results in a mixture of partially degraded organic matter, inorganic compounds, and microbial biomass [6].

Organic fertilizers offer the advantage of bonding nutrients to the organic matrix, limiting their leaching but also reducing their water solubility and availability for plants [7]. This imposes a long-term application, allowing environmental processes and management factors (such as precipitation, tillage, and irrigation) to modify the natural cycle of organic matter, driving the aggregate disruption and the nutrient redistribution [8]. To overcome this constraint, nutrients can be made available earlier using specific microorganisms or plant-derived substances that also exhibit growth-promoting activity. The application of biostimulants to plant leaves and seeds or to soil can stimulate root growth and help nutrient uptake and use efficiency [9], while increasing the beneficial microbial population [10,11].

The combined application of organic amendments and biostimulants can enhance soil health and productivity while reducing the supply of synthetic fertilizers and chemicals, hence promoting environmental sustainability and the circular economy [12], reducing harmful leaks and environmental pollution [13].

Eggplant (*Solanum melongena* L.) is an Asian native crop currently cultivated worldwide [14]. In Europe, Italy is the main producer of eggplant fruits, largely used in the Mediterranean diet, as they are low in calories and have high nutrient potential and are rich in chlorogenic acid and anthocyanin pigments, which are antioxidant compounds [15,16]. Deep and well-drained soils with a high organic matter content are essential for the root development of eggplant, and high levels of nitrogen (N) and phosphorus are required for the proper growth of the aerial part [17].

Based on the above stated principles, this paper delves into the agronomic implications of two organic amendments commonly used in Mediterranean agriculture—compost and digestate—combined with two different types of biostimulants, one derived from a tropical plant extract and one containing the beneficial microorganism *Trichoderma afroharzianum*, on eggplant fruit production and nutraceutical quality. To our knowledge, the synergistic effects of organic fertilization and biostimulation and its possible application to reduce the use of synthetic fertilizers have never been evaluated in eggplant cultivation.

## 2. Results

### 2.1. Plant Growth and Fruit Production

**Figure 1:**
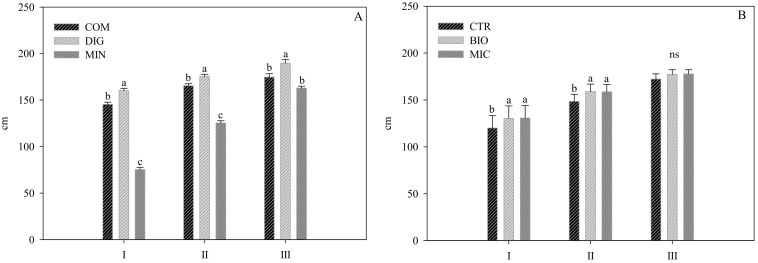
Plant height of eggplant plants in 3 different times of cycle (65, 107, and 139 DAT, corresponding to vegetative, productive, and senescence phases, namely I, II, and III, respectively) as affected by fertilization strategies (digestate—DIG; compost—COM; mineral—MIN) (**A**) and biostimulants application (plant-derived biostimulant—BIO; microbial biostimulant—MIC; not treated control—CTR) (**B**). Different letters indicate significant differences per *p* ≤ 0.05 according to Tukey’s test. ns—not significant. Vertical bars represent the standard error (n = 3).

The fertilization strategy affected the plant height, with digestate determining taller plants than the other treatments at all the measurement dates (Figure 1). Plant height showed a similar trend in the two biostimulant treatments, with higher values than the control (CTR) with both the microbial (MIC) and the plant-based (BIO) products, in the first two measurements (65 and 107 days after transplanting (DAT), corresponding to the vegetative growth and production stage, respectively), while no difference was observed in completely developed plants (139 DAT, senescence phase).

**Figure 2:**
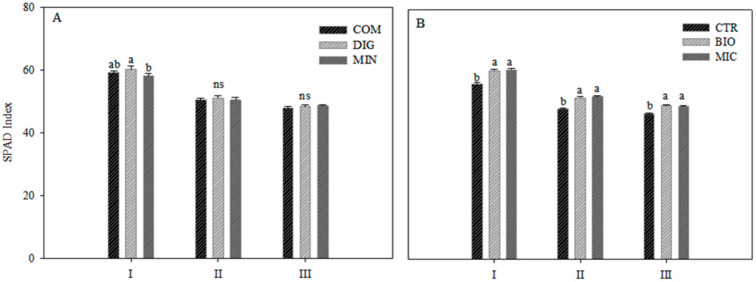
SPAD index in leaves of eggplant plants in 3 different times of cycle (65, 107, and 139 DAT, corresponding to vegetative, productive, and senescence phases, namely I, II, and III, respectively) as affected by fertilization strategies (digestate—DIG; compost—COM; mineral—MIN) (**A**) and biostimulants application (plant-derived biostimulant—BIO; microbial biostimulant—MIC; not treated control—CTR) (**B**). Different letters indicate significant differences per *p* ≤ 0.05 according to Tukey’s test. ns: not significant. Vertical bars represent the standard error (n = 3).

The leaf greenness, expressed as the SPAD index, showed a clear decrease over time (Figure 2). The fertilization strategy had a significant effect only at the first sampling (65 DAT, vegetative growth), with the highest value in digestate (Figure 2A) and the lowest in mineral treatment.

Biostimulants affected the SPAD index in all three measurement times, always determining higher values compared with CTR (Figure 2B).

The two experimental factors, fertilization strategy (F) and biostimulant application (B), did not show a significant interaction on the main parameters of fruit production; hence, Table 1 reports the average effects of each factor.

Fertilization affected all the main parameters of fruit yield and quality, except firmness (Table 1). The early marketable production (sum of the first five harvests) reached the highest value in plants fertilized with digestate (9.5 fruits m^−2^), followed by those treated with compost and minerals. The fruit weight was higher in digestate and mineral fertilization (+7% compared with compost).

In terms of total production, mineral fertilization gave the highest number of fruits (27.4 fruits m^−2^), while no difference occurred between the two organic treatments. The average fruit weight and dry matter (DM) percentage showed a significant difference only between mineral and compost, with the first giving heavier fruits (+6.7%). Differently, the nitrogen content was highest in DIG and MIN plants.

**Figure 3:**
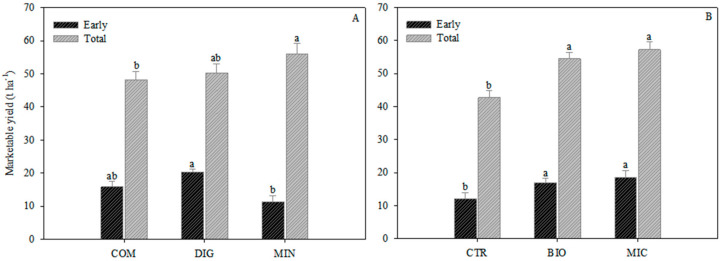
Early (sum of the first 5 harvests) and total (sum of all harvests) marketable yield of eggplant as affected by fertilization strategy (digestate—DIG; compost—COM; mineral—MIN) (**A**) and biostimulant application (plant-derived biostimulant—BIO; microbial biostimulant—MIC; not treated with biostimulant—CTR) (**B**). Different letters indicate significant different per *p* ≤ 0.05 according to Tukey’s test. Vertical bars indicate standard error (n = 3).

The biostimulant application increased the total number of fruits per plant compared with the control (Table 1), and determined better productive results, in terms of both early and total yield, with no difference between the two types of biostimulant (Figure 3B). Fruit firmness increased significantly only with the microbial biostimulant (+2.1% than CTR) (Table 1).

Fertilization strategies affected the fruit yield (Figure 3A), showing a better response of plants fertilized with digestate (about 20 t ha^−1^) in the early production, but better results in the mineral treatment (about 55 t ha^−1^) at the end of the cycle.

### 2.2. Eggplant Fruit Quality

#### 2.2.1. Color Parameters

Table 2 shows that no significant interaction between the two experimental factors was found in the fruit color, expressed as CIELAB color space parameters (L*, a*, and b*). The brightness parameter L* was unaffected by either the fertilization strategy or the biostimulant application. The colorimetric parameter a* (green to red component) was negatively influenced by the BIO biostimulant application compared with CTR. On the other hand, the colorimetric parameter b* (blue to yellow component) was influenced by both fertilization and the biostimulant, with the highest values in plants under DIG fertilization and untreated by the biostimulant, respectively.

#### 2.2.2. Antioxidant Activity and Main Nutraceutical Compounds

The interaction between the fertilization strategy and the biostimulant application also did not produce significant effects for qualitative traits.

The carotenoid content was not affected by the fertilization strategy, only by biostimulants, with a significant increase in the MIC treatment compared with the control (Table 3). The influence of fertilization was relevant only in the ascorbic acid (AsA) content and the hydrophilic antioxidant activity (HAA), with higher values under the organic fertilizers (Table 3). The biostimulant affected the phenol content and ABTS antioxidant activity, which were higher in CNTR and MIC treatments (Table 3).

## 3. Discussion

Intensive farming can reduce the content of organic matter and overall soil fertility, resulting in soil degradation [1]. Our study evaluated the agronomic outputs achieved using practices that increase the organic matter content in a loamy/sandy soil, in eggplant, a nutrient demanding crop, grown in a Mediterranean greenhouse. We studied the impact of two largely used amendments, a compost from urban solid residues and a solid digestate from livestock waste, in combination with two different types of biostimulants, one from tropical plant fermentation and one containing spores of the fungal biocontrol agent *Trichoderma afroharzianum* T-22.

As is known, eggplant thrives better in sandy loam and clay loam soils, with regular irrigation, ensuring constant soil moisture. Proper water management positively influences fruit yield and quality, and the microbial community linked to soil fertility [18]. Also, the plant genotype can influence the crop response to cultural practices such as fertilization. The ecotype “Napoletana”, tested in our experiment, has been used in two previous experiments [19,20], but none of them in combination with biostimulants to evaluate the environmental benefit related to the reduction in chemicals. Morra et al. [19] investigated plant response to different fertilization strategies, comparing two doses of compost from urban solid waste with inorganic fertilization. On the other hand, Pane et al. [20] applied eggplant extracts as a biocontrol agent against *Sclerotinia*.

Our results showed that the fertilization strategy influenced eggplant productivity. Both the organic fertilizers elicited higher early production, through a higher number of fruits per plant. However, mineral fertilization determined the highest total yield. This result could be due to the constant supply of nitrogen during the whole cycle, guaranteed by the application of minerals in fertigation. However, the average fruit weight did not differ statistically between mineral and digestate fertilization, presumably because of several reasons. For instance, the higher C/N ratio, which can prevent leaching and increase the fixation of N and the presence of functional elements (such as magnesium) in digestate, could have guaranteed sufficient N availability for the proper growth of the lower number of fruits [21,22,23].

Plant productive performance was enhanced by the application of biostimulants, as the plant-based product stimulates the plant primary metabolism [24], being rich in amino acids (the main carriers of organic nitrogen, used for protein synthesis) [25], and the microbial product increases the nutrient availability [7].

In our experiment, dry matter and N accumulation were influenced by fertilizers, with lower values in compost and no differences between digestate and mineral fertilizer. Dordas et al. [26] observed in various organs of maize a comparable dry matter production in plants grown with inorganic fertilizers and liquid manure. In contrast, in barley, Arduini et al. [27] obtained a higher N content and dry matter accumulation in plants fertilized with digestate, suggesting that these effects could be attributed to a supply of micronutrients and improved soil biological activity. On this topic, Dion et al. [28] reported that the nutritional composition of organic fertilizers affected microbial communities and their nitrogen mineralization rate.

Regarding fruit firmness, in their review, Rodrigues et al. [29] reported the effects of biostimulants in various examples on fruit plants (including vegetables), showing a varying response due to both the crop and the biostimulant product. Our results align with Consentino et al. [30], who observed a significant increase in fruit firmness due to the application of *T. afroharzianum* in grafted eggplant.

In the present study, the pigment content was influenced by the biostimulant application. Leaf greenness, expressed as the SPAD index, was higher in plants treated with both biostimulants, and a significant increase in carotenoid content was observed in the MIC treatment compared with the untreated control. *Trichoderma* is a fungal genus, which includes several species, widely employed in agriculture, alone and in consortium, as a biofertilizer, biostimulant, and bio-control agent [31]. The SPAD index is a non-destructive method for determining the health status of the plant through the indirect measurement of the chlorophyll and nitrogen content in leaves [32,33]. Our data show that it was positively affected by the biostimulant application but, to determine whether this is due to an effect on the chlorophyll content or nitrogen, further studies should be done. The scientific literature reports that the application of *Trichoderma* in consortium increased the carotenoid content in different plant species [34,35,36].

In our study, the type of fertilization influenced the amount of ascorbic acid and the hydrophilic antioxidant activity of eggplant fruits, which increased in plants grown with organic fertilization. Regarding the ascorbic acid in eggplant fruits, Michalojc and Buczkowska [37] reported that no differences occurred due to nitrogen sources, while Hassan et al. [38] showed a higher content under organic compared with mineral fertilizers, also hypothesizing that the composition of the organic fertilizer influences the plant metabolism and its interaction with soil. As mentioned, ascorbic acid has a hydrophilic antioxidant nature, so we can hypothesize that the HAA trend is dependent on ascorbic acid due to their high correlation [39]. It is known that compounds with antioxidant capacity, such as phenols, vitamins, and carotenoids, are an efficient countermeasure in the prevention of degenerative diseases [40,41], protecting biological systems against the harmful effects of oxidation [42]. In our experiment, the biosynthesis of phenols as well as the ABTS antioxidant activity were positively influenced by the biostimulant application. The positive effect of plant-based and microbial biostimulants on phenolic compounds was also observed in lettuce [43]. Concerning ABTS, we may hypothesize that its trend is triggered by the greater biosynthesis of carotenoids, as these compounds have a lipophilic behavior [16]; however, the specific mechanisms involved remain to be elucidated.

## 4. Materials and Methods

### 4.1. Plant Material, Growth Condition, and Experimental Treatments

The experiment was carried out in a plastic tunnel at the Department of Agricultural Sciences of the University of Naples Federico II (Portici, Italy; 40°49′ N, 14°15′ E, 72 m a.s.l.).

Seedlings of eggplant (*Solanum melongena* L.) ecotype “Napoletana” were transplanted on 22 February 2022, at the density of 2.1 plants m^−2^, in a loamy/sandy soil, with 1.69% organic matter, 0.11% total nitrogen (N), 97.3 ppm P_2_O_5_, and 1584.8 ppm K_2_O.

This study followed a 2-factor experimental design with 3 replicates, testing the combined effects of a fertilization strategy and biostimulant application. Three fertilization strategies were compared: compost (COM), the solid fraction of a digestate (DIG), and a traditional mineral fertilizer (MIN). The compost originated from the organic fraction of urban solid residues (Progeva s.r.l., Laterza, Taranto, Italy), containing 2% N (94.5% organic N) and 28.3% organic carbon, whereas the digestate, obtained by the anaerobic digestion of livestock waste, had 0.66% N (88% organic N) and 24.3% organic carbon (Power Rinasce S.p.A., Santa Maria la Fossa, Caserta, Italy). Fertilizer rates were determined based on the Fertilization Plain of Campania Region, to obtain the nitrogen dose for eggplant (280 kg ha^−1^). Accordingly, the compost was applied at 17 t ha^−1^ (fresh weight), while the digestate at 90 t ha^−1^ (fresh weight), both incorporated into the soil before transplanting. To avoid leaching, the mineral fertilizer was applied by a 10 fertigations using calcium nitrate (15.5% N). Based on the soil analyses, the P_2_O_5_ and K_2_O content were adequate to sustain the eggplant growth cycle; hence, no additional fertilizers were supplied.

The biostimulant treatments consisted of the plant-based biostimulant (BIO) and the microbial biostimulant (MIC). BIO (Auxym^®^, Italpollina S.p.A., Rivoli Veronese, Italy) was obtained by fermenting tropical plants such as hibiscus (*Hibiscus* spp. L., 1753), and applied 4 times as a foliar spray, starting on February 28. MIC (Trianum-P^®^, Koppert Biological Systems, Berkel en Rodenrijs, The Netherlands) contained the microorganism *Trichoderma afroharzianum* (ex *T. harzianum*) strain Rifai KRL-AG2 (T-22) at a minimum concentration of 1 × 10^9^ CFU g^−1^. MIC was applied 5 times: the 1st one as root immersion (at the dose of 2.5 kg ha^−1^) on the day of transplant, followed by 4 soil applications (at the dose of 1.0 kg ha^−1^). No biostimulant was applied in the control (CNT). The experiment consisted of 27 plots (3 fertilization strategies × 3 biostimulant treatments × 3 replicates).

**Figure 4:**
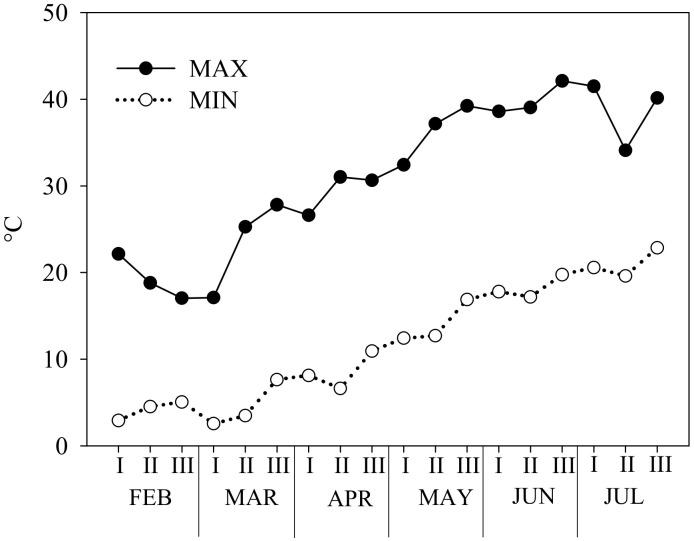
Trend of minimum and maximum temperatures recorded in the greenhouse during the experiment. I, II, and III indicate the 10-day intervals of each month.

The air temperature in the tunnel was measured by dataloggers (Vantage Pro2, Davis Instruments, Hayward, CA, USA) distributed randomly and located 30 cm above the canopy. The air temperature (minimum and maximum) trend is shown in Figure 4.

### 4.2. Biometric Measurements, Leaf Greenness, and Fruit Yield

Three times during the growth cycle, the height (cm) of eggplant plants was measured, specifically at 65, 107, and 139 days after transplanting (DAT, and corresponding to vegetative, full production, and senescence phases, respectively), starting from the bottom until the shoot tip.

On the same days as height measurement, soil plant analysis development (SPAD) was also performed using a portable Konica Minolta chlorophyll meter (SPAD-502, Tokyo, Japan). Measurements were carried out on the fifth fully expanded leaf starting from the apex, using three plants per repetition.

The harvests were carried out 14 times, starting on May 19 until July 21 (the last one), on the fruits that reached marketable size. We considered the fruits harvested during the first five harvests as early, while the sampled fruits from the eighth harvest were frozen for the following qualitative analysis.

At each harvest, the number and the average weight of fruits were measured and used to calculate the yield, while a subsample was collected, weighted, and dried in an air oven at 72 °C until constant weight to determine the dry matter (DM) percentage.

### 4.3. Qualitative Traits

#### 4.3.1. Color Parameters and Firmness

The color parameters, expressed as stated by Commission International de l’eclairage (CIELAB) L* (brightness), a* (redness component), and b* (yellowness component), were measured using a CR-300 Chroma Meter (Minolta Camera Co., Ltd., Osaka, Japan) in the middle portion of eggplant fruits at each harvest, in 4 points around the fruit circumference.

On the same fruits, the firmness was measured, expressed in kg cm^−2^, using a digital penetrometer (T.R. Turoni s.r.l., Forlì, Italy) equipped with an 8 mm diameter probe.

#### 4.3.2. Nitrogen and Carotenoid Content

The frozen eggplant fruits of the eighth harvest were used for various determinations, such as the nitrogen content (expressed in percentage of dry weight) by the Kjeldahl method [44] after sample drying.

Moreover, a portion (1 g) of frozen fruits was extracted in pure acetone and centrifugated at 3000 G (for 5 min). Subsequently, the absorbance of the supernatant was measured with a Hach DR 2000 spectrophotometer (Hach Co., Loveland, CO, USA) at 470 nm to determine the carotenoid content [45].

#### 4.3.3. Ascorbic Acid and Total Phenol Content, and Antioxidant Activity

The frozen fruits were lyophilized and used to determine the following analyses. The ascorbic acid (AsA) assay was performed according to the protocol proposed by Kampfenkel et al. [46], using a spectrophotometer. It was expressed as mg g^−1^ fw. Two hundred mg of frozen dried samples were extracted by two different procedures to assess the eggplant fruit’s antioxidant capacity. The first occurred with distilled water to determine the hydrophilic fraction and was measured using the method proposed by Fogliano et al. [47], involving N, N-dimethyl-p-phenylenediamine (DMPD), while the lipophilic fraction was extracted by methanol and the extract was measured by the 2,20-azinobis 3-ethylbenzothiazoline-6-sulfonic acid ABTS method [48]. Both antioxidant activities were determined by UV-Vis spectrophotometry at the absorbance of 505 and 734 nm, respectively. Hydrophilic and lipophilic fractions were expressed as mmol ascorbic acid and Trolox (6-hydroxy-2,5,7,8-tetramethylchro man-2-carboxylic acid) per 100 g^−1^ dw, respectively.

Total phenolics were quantified in methanol extracts using the Folin–Ciocalteu procedure [49], and the absorption was measured at 765 nm using the same spectrophotometer. The result was expressed as mg gallic acid per g dw.

### 4.4. Statistical Analysis

SPSS 2022 software v. 22.0 (IBM SPSS Inc., Chicago, IL, USA) was used for statistical data analysis. A two-way ANOVA was conducted to evaluate the effects of fertilization (F) and biostimulant (B) factors in a two-factorial experimental design (with three replicates per treatment). Additionally, a one-way ANOVA was used to compare the mean effects of individual factors. Significant statistical differences were determined by Tukey’s HSD test at the level of *p* ≤ 0.05.

## 5. Conclusions

Our results highlight that, in eggplants, fertilization with digestate allowed to reach a total yield consistent with that obtained with mineral fertilizers. Interestingly, both the digestate and compost elicited early production compared with mineral fertilization, with consequent economic advantage for farmers, since early fruits are usually worth more than the ordinary ones. Also, organic fertilizers improved some qualitative traits of eggplant fruits.

Biostimulant treatments always determined better productive performance compared with non-treated plants, along with a superior product quality, particularly when the microbial-based inoculant was used.

In conclusion, the substitution of mineral fertilizers with digestate and the use of both plant-based and microbial biostimulants are sustainable practices in eggplant cultivation, contributing to maintaining a proper soil nutrient balance without detrimental effects on yield, and possible improvement on fruit quality and useful economical outputs due to the higher early production.

## Figures and Tables

**Table 1:** Number and average fresh weight of eggplant fruits of early and total marketable production, dry matter (DM) percentage, firmness, and N-Kjeldahl content, as affected by fertilization strategy (compost (COM), digestate (DIG), mineral (MIN)), and biostimulant application (untreated control (CTR), plant-based biostimulant (BIO), microbial biostimulant (MIC)).

Treatments	Early Marketable Yield		Total Marketable Fruits		DM	Firmness	N-Kjeldahl
	n° m^−2^	g fruit^−1^	n° m^−2^	g fruit^−1^	%	kg cm^−2^	%
**Fertilization**							
COM	7.8 ± 0.7 b	199.5 ± 3.9 b	25.5 ± 1.3 b	186.2 ± 4.7 b	6.53 ± 0.1 b	1.89 ± 0.02	1.92 ± 0.04 b
DIG	9.5 ± 0.7 a	213.5 ± 3.8 a	25.9 ± 1.2 b	193.4 ± 2.3 ab	6.87 ± 0.1 ab	1.89 ± 0.02	2.08 ± 0.02 a
MIN	5.3 ± 0.3 c	213.5 ± 6.8 a	27.4 ± 1.1 a	198.7 ± 2.6 a	7.14 ± 0.1 a	1.90 ± 0.02	1.98 ± 0.07 ab
**Biostimulant**							
CTR	6.0 ± 0.6 b	200.3 ± 4.8 b	22.2 ± 0.7 b	189.3 ± 1.9	6.73 ± 0.15	1.84 ± 0.01 b	1.95 ± 0.05
BIO	8.1 ± 0.6 a	208.9 ± 5.9 ab	27.7 ± 0.6 a	193.3 ± 2.6	6.91 ± 0.12	1.90 ± 0.01 ab	2.01 ± 0.04
MIC	8.5 ± 0.9 a	217.3 ± 4.1 a	28.8 ± 0.8 a	195.7 ± 2.5	6.91 ± 0.11	1.94 ± 0.02 a	2.02 ± 0.05
**Significance**							
Fertilization (F)	**	*	**	*	**	ns	*
Biostimulant (B)	**	*	**	ns	ns	**	ns
F × B	ns	ns	ns	ns	ns	ns	ns

Within each column, different letters indicate significant differences according to Tukey’s test at *p* ≤ 0.05; ns, *, and **: not significant and significant at *p* < 0.05 and *p* < 0.01, respectively. Values are mean ± standard error (n = 3).

**Table 2:** Color parameters (L*: brightness, ranging from 0 = black to 100 = white; a*: chroma component from green (−60) to red (+60); b*: chroma component from blue (−60) to yellow (+60)) in eggplant fruits as affected by fertilization strategy (compost (COM), digestate (DIG), mineral (MIN)) and biostimulant application (untreated control (CTR), plant based biostimulant (BIO), microbial biostimulant (MIC)).

Treatments	L*	a*	b*
**Fertilization**			
COM	25.7 ± 0.31	7.21 ± 0.58	0.96 ± 0.17 ab
DIG	26.3 ± 0.44	7.38 ± 0.40	1.07 ± 0.32 a
MIN	25.7 ± 0.18	7.22 ± 0.45	0.60 ± 0.09 b
**Biostimulant**			
CTR	26.5 ± 0.46	8.26 ± 0.36 a	1.40 ± 0.28 a
BIO	25.5 ± 0.22	6.31 ± 0.23 b	0.51 ± 0.10 b
MIC	25.7 ± 0.17	7.24 ± 0.53 ab	0.71 ± 0.10 b
**Significance**			
Fertilization (F)	ns	ns	*
Biostimulant (B)	ns	*	**
F × B	ns	ns	ns

Within each column, different letters indicate significant differences according to Tukey’s test; ns, *, and ** not significant or significant at *p* < 0.05 and *p* < 0.01, respectively.

**Table 3:** Main bioactive compounds and antioxidant activity in eggplant fruits affected by fertilization strategy (compost (COM), digestate (DIG), mineral (MIN)) and biostimulant application (untreated control (CTR), plant-based biostimulant (BIO), microbial biostimulant (MIC)).

Treatments	Carotenoids	AsA	Phenols	HAA	ABTS
	µg g^−1^ fw	mg g^−1^ fw	mg Gallic Acid g^−1^ dw	mmol AA 100 g^−1^ dw	mmol Trolox 100 g^−1^ dw
**Fertilization**					
COM	0.017 ± 0.001	45.42 ± 5.45 a	2.80 ± 0.09	5.26 ± 0.35 ab	12.87 ± 0.51
DIG	0.019 ± 0.001	32.82 ± 5.06 ab	2.44 ± 0.16	5.71 ± 0.28 a	13.11 ± 0.65
MIN	0.020 ± 0.002	29.15 ± 3.61 b	2.73 ± 0.17	4.88 ± 0.14 b	12.47 ± 0.80
**Biostimulant**					
CTR	0.016 ± 0.001 b	36.87 ± 4.77	2.62 ± 0.08 ab	5.38 ± 0.32	12.81 ± 0.69 ab
BIO	0.018 ± 0.001 ab	37.44 ± 5.46	2.46 ± 0.17 b	5.25 ± 0.26	12.11 ± 0.58 b
MIC	0.022 ± 0.002 a	33.08 ± 5.72	2.89 ± 0.16 a	5.22 ± 0.30	13.53 ± 0.65 a
**Significance**					
Fertilization (F)	ns	*	ns	*	ns
Biostimulant (B)	*	ns	*	ns	*
F × B	ns	ns	ns	ns	ns

Within each column, different letters indicate significant differences according to Tukey’s test; ns, and * not significant or significant at *p* < 0.05, respectively. AsA—ascorbic acid; HAA—hydrophilic antioxidant activity; ABTS—antioxidant activity; dw—dry weight; fw—fresh weight. Analyses were performed on samples from the 8th harvest. Values are mean (n = 3) ± standard error.

## Data Availability

The original contributions presented in this study are included in the article. Further inquiries can be directed to the corresponding author.
